# *Eleucine indica* Possesses Antioxidant, Antibacterial and Cytotoxic Properties

**DOI:** 10.1093/ecam/nep091

**Published:** 2011-06-05

**Authors:** Adel S. Al-Zubairi, Ahmad Bustamam Abdul, Siddig Ibrahim Abdelwahab, Chew Yuan Peng, Syam Mohan, Manal Mohamed Elhassan

**Affiliations:** ^1^Laboratory of Cancer Research MAKNA-UPM, Institute of Biosciences (IBS), Universiti Putra Malaysia, Serdang, Selangor DE 43400, Malaysia; ^2^Department of Biochemistry and Molecular Biology, Faculty of Medicine and Health Sciences, University of Sana'a, Sana'a, Yemen; ^3^Department of Biomedical Sciences, Faculty of Medicine & Health Sciences, Universiti Putra Malaysia, Serdang, Selangor DE, Malaysia

## Abstract

The use of evidence-based complementary and alternative medicine is increasing rapidly. *Eleucine indica* (EI) is traditionally used in ailments associated with liver and kidneys. The therapeutic benefit of the medicinal plants is often attributed to their antioxidant properties. Therefore, the aim of this study was to screen the hexane, dicholoromethane, ethyl acetate (EA) and methanol extracts (MeTH) of EI for their antioxidant, antibacterial and anti-cancer effects using total phenolic contents (TPCs) and DPPH, disc diffusion method and MTT cytotoxicity assays, respectively. The MeTH was showed to have the highest TPC and scavenging activity (77.7%) on DPPH assay, followed by EA (64.5%), hexane (47.19%) and DCM (40.83%) extracts, whereas the MeTH showed no inhibitory effect on all tested bacteria strains. However, the EA extract exhibited a broad spectrum antibacterial activity against all tested bacteria except *Bacillus subtilis*, in which this bacterium was found to be resistant to all EI extracts. Meanwhile, hexane extract was demonstrated to have a remarkable antibacterial activity against methicillin resistant *Staphylococcus aureus* (MRSA) and *Pseudomonas aeruginosa*, while the dicholoromethane extract did not exhibit significant activity against *P. aeruginosa*. None of the extracts showed significant cytotoxic activity towards MCF-7, HT-29 and CEM-SS human cancer cell lines after 72 h incubation time (IC_50_ > 30 *μ*g/ml). These results demonstrate that the extract prepared from the EI possesses antioxidant activity *in vitro* in addition to antibacterial properties. Further investigations are needed to verify the antioxidant effects *in vitro* and *in vivo*.

## 1. Introduction

*Eleucine indica* (*Poaceae*) (EI) or goosegrass, generally considered an adventitious species, is native in the tropics and subtropical regions [1]. Goosegrass has a broad tolerance to a wide range of environmental conditions, but its vegetative growth is significantly reduced during dry seasons [2, 3]. The whole plant, especially the root, is depurative, diuretic, febrifuge and laxative, and hence is used for the treatment of influenza, hypertension, oliguria and urine retention. The plant has been the component of “basic remedy” in Vietnamese traditional medicine [2, 3] and also used for kidney problems in Trinidad and Tobago [4]. The seed is sometimes used as a famine food and also used in the treatment of liver complaints. Many herbal products, such as the one studied in this article, have traditional uses that are now being investigated to create an evidence base that will facilitate their inclusion in general medical practice.

Plants produce a higher number of naturally occurring secondary metabolites, many of them with unique pharmacologic activities. These metabolites include the flavonoids, phenols and phenolic glycosides, saponins, cyanogenic glycosides, unsaturated lactones and glucosinolates [5–8]. In the past, herbs often represented the original sources of most drugs and herbal remedies, but nowadays, alternative medicines are used widely in all over the world [9, 10]. Herbal-derived remedies need a powerful and deep assessment of their pharmacological qualities [11]. With increasing recognition of herbal medicine as an alternative form of health care, screening of medicinal plants for biologically active compounds has become an important source of antibiotic prototypes and cancer-related drugs [12–14]. Hence, for selecting crude plant extracts with potential useful properties, *in vitro* screening methods have been used for further in-depth chemical elucidation and pharmacological investigations [15]. To date, few studies of EI have been reported; specially, its phytochemical content of sterol glucosides forms [16] and C-glycosylflavone having anti-inflammatory activity [17]. To our knowledge, no other scientific investigation has been reported in evaluating EI's therapeutic potential. Therefore, this study was conducted to evaluate the antioxidant, antibacterial and anti-cancer activities of the hexane, dichloromethane (DCM), ethyl acetate (EA) and methanol extracts (MeTH) of EI using total phenolic content (TPC), DPPH, disc diffusion and MTT cytotoxicity assay methods of analysis.

## 2. Methods

### 2.1. Collection of Plant Materials

EI leaves were collected in July 2007 from the Laboratory of Natural Products, Institute of Bioscience, UPM, Selangor, Malaysia. Authentication was done at the same laboratory where the voucher specimen (EI-L100158) was deposited.

### 2.2. Extraction Procedure

The leaves were air dried and then oven dried at reduced temperature and then ground into powder before cold maceration. The powdered leaves (300 g) were extracted with different solvents in the order of increasing polarity. The solvents used were hexane, DCM, EA and methanol. Extractions were done for 7 days with occasional shaking and the process repeated three times. The residue was air dried overnight and used for next solvent extraction (DCM) as per the above procedure and the same procedure was repeated for next two other solvents (EA and methanol). Finally, the combined extracts for each solvent were filtered through Whatman No. 41 filter paper (pore size 20–25 *μ*m) and dried under vacuum using a rotary evaporator and kept at 4°C until required.

### 2.3. Antioxidant Assays

#### 2.3.1. Amount of Total Phenolic Compounds

TPC of the EI extracts was determined using Folin-Ciocalteu reagent method [18]. Briefly, stock solutions of EI extracts were prepared in a concentration of 20 mg/ml. Fifty microliters of this solution was transferred to test tubes (*n* = 3). To this, 0.4  ml of Folin-Ciocalteu reagent (1 : 10) was added and mixed thoroughly. After 1 min, 0.8 ml of sodium bicarbonate solution (NaHCO_3_ 7.5%) was added and the mixture was allowed to stand for 30 min with intermittent shaking. Absorbance was measured at 765 nm using a Shimadzu UV-Vis spectrophotometer. The TPC was expressed as gallic acid equivalents (GAE) in mg/g extract, obtained from the standard curve of gallic acid solutions. The gallic acid standard curve was established by plotting concentration (mg/ml) versus absorbance (nm) (*y* = 5.145*x*+0.014; *R* ^2^ = 0.9975), where *y* is absorbance and *x* is concentration.

#### 2.3.2. DPPH Radical Scavenging Assay

The radical scavenging activity of extracts was determined following the method of Changwei et al. [19] with slight modification. The radical scavenging activity of the extracts against stable 2,2-diphenyl-2-picrylhydrazyl hydrate (DPPH, Sigma-Aldrich Chemie, Steinheim, Germany) was determined spectrophotometrically. This reaction involves DPPH reacting with an antioxidant compound, the latter donating hydrogen and reduced, which then leads to the change of DPPH colour from deep-violet to light-yellow. This colour change was monitored at 517 nm wavelength. We selected the DPPH free radical scavenging assay due to its straightforwardness, quickness, sensitivity and reproducibility [20]. Apart from this, the assay is useful to screen large number of samples with different polarity.

Briefly, EI extracts stock solutions were prepared at 100 mg/ml in ethanol. Since the MeTH was not fully soluble in ethanol (even after sonication for 5 min), the extract was dissolved in dimethyl sulfoxide (DMSO). The working solution was prepared in methanol at concentration of 500 *μ*g/ml. The DPPH solution in methanol (2.5 mg/ml) was freshly prepared before all absorption measurements. Five microliter of this solution was mixed with 100 *μ*l extract solutions in microtiter 96-well plates. The samples were kept in the dark for 30 min at an ambient temperature and the decrease in absorption was measured. Absorption of blank sample containing the same amount of methanol and DPPH solution was prepared and measured daily. The experiments were done in triplicates.

The radical scavenging activity was calculated by the following formula:

(1) $$\text{Percentage}\;\text{inhibition}=\frac{\left(A_{\text{B}}-A_{\text{A}}\right)}{A_{\text{B}}}\times 100,$$

where *A*_B_ is absorption of blank sample (*t* = 0 min), *A*_A_ is absorption of tested extract solution (*t* = 30 min). A commercial antioxidant, butylated hydroxytoluene (BHT), was used as the reference standard.

### 2.4. Antimicrobial Activity of EI

#### 2.4.1. Bacterial Strains

The antimicrobial activity of plant extracts was evaluated using two Gram-positive bacteria, methicillin resistant *Staphylococcus aureus* (MRSA) and *Bacillus subtilis* B29, and other two Gram-negative bacteria, *Pseudomonas aeruginosa* 60690 and *Salmonella choleraesuis*. All the bacterial strains were obtained from the Laboratory of Molecular Biomedicine, Institute of Bioscience, Universiti Putra Malaysia, Serdang, Malaysia.

#### 2.4.2. Antibacterial Assay

Screening of antibacterial activity was carried out by determining the zone of inhibition using the Paper Disc (6 mm in diameter, Whatman No. 1) Diffusion method [21, 22]. The procured microorganism strains were inoculated in a Petri dish containing nutrient broth at 37°C for 24 h and were referred as seeded broth. The density of bacterial suspension of the cultures was standardized turbidometrically to 500 000–1 000 000 colony forming units per milliliter (CFU/ml) at wavelength of 600 nm. The EI extracts were dissolved initially in DMSO, diluted to a concentration of 100 mg/ml and filtered using 0.45 *μ*m millipore filters. Sterile discs were impregnated with the extract solutions (0.05 ml from 100 mg/ml extract) and placed in inoculated agar. Streptomycin (10 *μ*g/disc) was used as the standard. Controls were prepared using similar solvents but without extracts. The inoculated plates containing both the test and standard discs were incubated at 37°C for 24 h.

### 2.5. Cytotoxicity Assay

#### 2.5.1. Preparation of Extracts

To screen the extracts of EI, dried extracts of the plant were dissolved in 1 ml of DMSO to give a stock solution of extract at 10 mg/ml. All extracts were kept at 4°C throughout the experiments. Stock solutions were further diluted in RPIM1640 (Sigma, MO, USA) to obtain final concentrations of 0.469, 0.938, 1.875, 3.75, 7.5, 15 and 30 *μ*g/ml.

#### 2.5.2. Cell Culture Condition

MCF-7 human breast cancer cells and HT-29 human colon carcinoma cells were purchased from the American Type Culture Collection (ATCC), USA. Human T4-lymphoblastoid cell line, CEM-SS, used in this study were obtained from The NIH AIDS Research and Reference Reagent Program, USA. The cancer cells were grown at 37°C in humidified CO_2_ incubator with 5% CO_2_ in RPMI-1640 media supplemented with 10% fetal bovine serum (Invitrogen Corp., Auckland, New Zealand).

#### 2.5.3. Cell Growth Inhibition Assay

Cell suspension (5 × 10^5^ cells/ml) was plated out into 96-well microtiter plate. Plant extracts were initially dissolved in DMSO as mentioned earlier, with the final concentration of DMSO being 0.1% (v/v). Serial dilutions of the sample were prepared in RPIM1640. The cytoxicity profiles of the extracts were assessed using 3-[4,5-dimethylthiazol-2-yl]-2,5-diphenyltetrazolium bromide (MTT) microculture tetrazolium viability assay as described by Mosmann [23]. Thereafter, various concentrations of the plant extract samples were plated out in triplicates. Each plate included untreated cell controls and a blank cell-free control. After 68 h of incubation, MTT (5 *μ*g/ml) was added to each well and re-incubated for further 4 h. Then, the media was removed and DMSO was added into each well to solubilize the formazan crystals. Finally, the absorbance was read at wavelength of 595 nm using a microtitre plate reader (Labsystems iEMS Reader MF) and the percentage cell viability was calculated with the appropriate controls taken into account. The concentration which inhibited 50% of cellular growth (IC_50_ value) was determined and the inhibitory rate of cell proliferation was calculated by the following formula:

(2) $$\text{Growth}\;\text{inhibition}=\frac{{\text{OD}}_{control}-{\text{OD}}_{\text{treated}}}{{\text{OD}}_{control}}\times 100,$$

where OD is the optical density.

Cytotoxicity of the sample towards the cancer cells was expressed as IC_50_ values (i.e. the EI extract concentration reducing the absorbance of treated cells by 50% in respect to untreated cells).

### 2.6. Statistical Analysis

Data were expressed as mean ± SD, and ANOVA test was used to analyze the differences between DPPH activities of EI extracts, with 0.05 as a level of significance. Ordinary least squares regression was used to calculate the standard curve of gallic acid. Pearson correlation coefficient was used to measure the relationship between DPPH, scavenging activity and TPC.

## 3. Results

### 3.1. Antioxidant Assays

**Figure 1:**
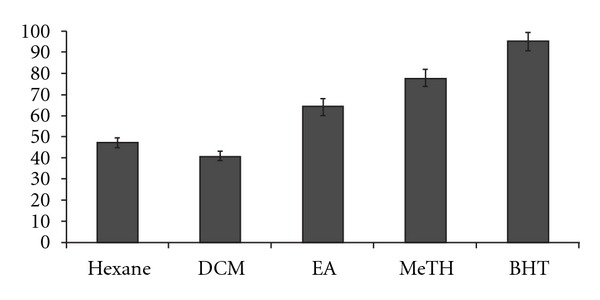
DPPH absorption inhibition (%) of EI extracts isolated with methanol, EA, hexane and DCM. Data represent mean ± SD, *n* = 3.

Total phenolic content was determined using Folin-Ciocalteu reagent and the results revealed that the MeTH of EI having the highest TPC (450 ± 210 GAE mg/g extract; *P* < .05; Table 1) when compared with DCM, hexane and EA extracts. Meanwhile, in the DPPH inhibition results (Figure 1), the MeTH has shown to have the most free radical scavenging activity as observed from the percentage inhibition of the DPPH absorption (77.7%). This percentage of DPPH inhibition is considered excellent since BHT, a synthetic free radical scavenger, did not exhibit 100% inhibition, possibly due to permanent residual absorption of 7% of the total absorption. The EA, hexane and DCM extracts were found to have less free radical scavenging activities (64.5, 47.19 and 40.83%, resp.) when compared with the MeTH. These results reveal that there is a strong and significant correlation between TPC and DPPH free radical scavenging activity of the EI extracts (Pearson correlation coefficient = 0.507; *P*< .05).

### 3.2. Antimicrobial Activity of EI

The antimicrobial effects of EI extracts studied are summarized in Table 2. The antibacterial activities of the different extracts of EI were evaluated using both Gram-positive and Gram-negative bacteria. The highest antibacterial activity observed was obtained by the hexane extract on MRSA, while the DCM extract showed weak activity on *P. aeruginosa*. On the other hand, the EA extract showed a broad spectrum activity, compared with the positive control (Streptomycin) against all tested bacteria except for *B. subtilis*, which showed resistance to all extracts of EI. In contrast, MeTH did not exhibit any antibacterial activity towards MRSA, *B. subtilis* B29, *P. aeruginosa* 60690 and *S. choleraesuis*. The positive control, Streptomycin, had shown zones of inhibition of 20 ± 1.5, 20 ± 1.3, 23 ± 1.5 and 23 ± 1.0 mm in MRSA, *P. aeruginosa*, *S. choleraesuis and B. subtilis*, respectively.

### 3.3. Anti-Tumor Effects of EI Extract

The cytotoxic effects of the extracts of EI on MCF-7, HT-29 and CEM-SS cancer cell lines were examined in this study by MTT. A dose-response curve for the percentages of viability cells (0–100%) was plotted against concentrations of 0.469–60 *μ*g/ml of the extracts. The four extracts did not produce any cytotoxic effects on MCF-7, HT-29 and CEM-SS cancer cell lines (>30 *μ*g/ml) when compared with the control RPMI 1640 and DMSO.

## 4. Discussion

Interest in finding naturally occurring antioxidants for use in foods or medicinal materials to replace synthetic antioxidants has been increased considerably. Restrictions on the use of synthetic antioxidants such as butylated hydroxyanisole (BHA) and BHT are being imposed due to their carcinogenicity [24]; therefore, a need for identifying alternative natural and safe sources of antioxidants, especially of plant origin, has increased in recent years [25]. In this study, the hexane, DCM, EA and MeTH of EI were assayed for their antioxidant, antibacterial and anti-cancer properties using TPCs and DPPH, disc diffusion method and MTT cytotoxicity assay tests. The extraction procedure used was cold maceration, in which the solvents used were in the order of increasing polarity.

The therapeutic benefit of medicinal plants is usually contributed to their antioxidant properties [26–28]. Phenolic compounds possess diverse biological activities such as anti-inflammatory, anti-carcinogenic and anti-atherosclerotic activities. These activities might be related to their antioxidant activity [29]. Other studies showed that there were significant correlations between phenolic compounds and antioxidant properties of medicinal plants under investigations [30, 31]. In this respect, a part of our study was determining TPC in different extracts obtained from EI. Our results revealed that there is a strong and significant correlation between TPC and DPPH free radical scavenging activity of the EI extracts. As a result, the MeTH of EI was showed to have the highest TPC when compared with DCM, hexane and EA extracts. Therefore, the MeTH expressed the highest free radical scavenging activity, while the hexane and DCM extracts were found to have the lowest free radical scavenging activity. However, the antioxidant properties of the MeTH mostly attributed to the phenolic composition, in contrast to the other fractions in which they showed to have less amount of phenolic content as well as less free radical scavenging activities. On the other hand, the lower antioxidant activity of DCM with higher TPC compared with EA may be explained by the less polar components of this fraction than the EA. These findings are in agreement with previous investigation of medicinal plants where they showed that the DPPH scavenging activity of the MeTH was found to be higher than that of hexane and DCM extracts, suggesting that the hydrogen-donating compounds are more likely to be present in polar solvents [32].

In addition to screening the antioxidant properties, we have also investigated the antibacterial activities of different extracts from EI (Table 2). Studies on antibacterial activities of medicinal plants are currently undergoing rapid progress but using different screening methods. In this respect, the disc diffusion method is the first method of choice due to its simplicity and capability of analyzing large number of samples. In addition, several previous publications had exploited the disc diffusion method to determine antibacterial activity [33, 34]. Results of the present study indicated that hexane extract is the good bactericidal fraction of EI towards the tested bacterial strains, MRSA, *B. subtilis* B29, *P. aeruginosa* 60690 and *S. choleraesuis*, while EA extract was the next in potency as the antibacterial agent. However, the results of antibacterial activities of EI extracts indicated that MeTH did not exhibit any antibacterial activity. Hence, hexane extract will be the best target for further research for the development of antibacterial agents.

Cytotoxicity assay results revealed that the hexane, DCM, EA and MeTH of EI were found to have IC_50_ values of over 30 *μ*g/ml against MCF-7, HT-29 and CEM-SS cancer cell lines after 72 h incubation. Thus, the four extracts of EI were conferred non-effective in inducing cell death towards the cancer cell lines MCF-7, HT-29 and CEM-SS, following guidelines of the American National Cancer Institute. It has been reported previously that some plant extracts have shown to have multi-biological activities of possessing both antibacterial and antioxidant activities [35–37]. In this respect, our study of the EI extracts has demonstrated such multi-biological activities to exist, suggesting the presence of chemical constituents in EI responsible for such behavior. These results suggest that the EI possess antioxidant properties and could be used as alternative natural antioxidants. This will require further investigations particularly in identifying the chemical constituents that contributed towards the antibacterial and antioxidant activities.

## Figures and Tables

**Table 1:** TPC in EI extracts.

Extract	TPC as GAE (mg/g extract)
Hexane	210 ± 34^b^
DCM	310 ± 83^b^
EA	175 ± 12^a^
MeTH	450 ± 210^c^

Each value in the table is represented as mean ± SE (*n* = 3); different letters in the same column indicate significant difference (*P*< .05).

**Table 2:** Paper disk diffusion of EI extracts effects on bacterial growth.

EI extracts	Bacterial strains			
	Diameter of inhibition (mm)			
	MRSA	PA	SC	BS
Hexane	13 ± 0.9	11 ± 0.8	−	−
DCM	−	7 ± 0.4	−	−
EA	10 ± 0.7	12 ± 0.5	11 ± 0.7	−
MeTH	−	−	−	−
Streptomycin (10 *μ*g/disc)	20 ± 1.5	20 ± 1.3	23 ± 1.5	23 ± 1.0

The screening of the extracts antibacterial effect was carried out by determining the zone of inhibition using paper disc (6 mm in diameter, Whatman No. 1) diffusion method (*n* = 2). MRSA, methicillin resistant *Staphylococcus aureus*; PA, *Pseudomonas aeruginosa*; SC, *Salmonella choleraesuis* and BS, *Bacillus subtilis*. Streptomycin showed 20, 20, 23 and 23 mm inhibition towards MRSA, PA, SC and BS, respectively.
